# Grifolin Exhibits Promising Antimicrobial Potential Against MRSA Infection

**DOI:** 10.3390/biology15212019

**Published:** 2026-11-06

**Authors:** Guosheng Hu, Yun Zhou, Mengxi Wang, Rong Huang, Jikai Liu

**Affiliations:** The Modernization Engineering Technology Research Center of Ethnic Minority Medicine of Hubei Province, School of Pharmaceutical Sciences, South-Central Minzu University, Wuhan 430074, China

**Keywords:** grifolin, methicillin-resistant *Staphylococcus aureus*, antibacterial activity, biofilm eradication, diabetic wound

## Abstract

Methicillin-resistant *Staphylococcus aureus* (MRSA) exhibits widespread multidrug resistance and frequently causes refractory wound infections, particularly in diabetic patients, creating an urgent need for new therapeutic options. Grifolin, a naturally occurring phenolic compound with antibacterial, anti-inflammatory, and antioxidant activities, represents a promising candidate for the comprehensive treatment of infected diabetic wounds. Herein, we systematically investigated the antibacterial activity and mechanism of grifolin against MRSA, as well as its therapeutic efficacy in a murine model of MRSA-infected diabetic wounds. In vitro, grifolin exhibited potent antibacterial activity against MRSA (MIC = 3 μg/mL, MBC = 4 μg/mL), effectively eliminated mature biofilms, and potentiated conventional antibiotics. Integrated transcriptomic and proteomic analyses, corroborated by qRT-PCR, revealed coordinated changes in membrane integrity, translation and metabolism, and quorum sensing and virulence. In vivo, grifolin significantly reduced the wound bacterial burden, suppressed the expression of pro-inflammatory cytokines IL-6 and TNF-α, promoted angiogenesis and tissue remodeling, and accelerated diabetic wound healing with favorable preliminary tolerability. This study highlights grifolin as a promising lead compound for combating MRSA infections and treating associated diabetic wound complications.

## Simple Summary

Methicillin-resistant *Staphylococcus aureus* (MRSA) is a major cause of difficult-to-treat infections due to its increasing antibiotic resistance and ability to form protective biofilms. The discovery of safe and effective alternatives to conventional antibiotics is therefore urgently needed. In this study, we investigated grifolin, a natural phenolic compound isolated from an edible fungus, as a potential anti-MRSA agent. Grifolin showed strong antibacterial activity, eradicated biofilms, and enhanced the effectiveness of common antibiotics. In mice with MRSA-infected diabetic wounds, it reduced bacterial infection, inhibited inflammation and improved wound healing with good safety. These findings suggest that grifolin may serve as a promising natural compound for developing new strategies against drug-resistant bacterial infections.

## 1. Introduction

The global spread of antimicrobial resistance has become one of the most critical public health threats of the 21st century. Among drug-resistant pathogens, MRSA is a leading cause of both hospital-acquired and community-associated infections [1]. MRSA has acquired resistance to most β-lactam antibiotics, chiefly through the mecA-encoded penicillin-binding protein 2a (PBP2a), and frequently displays multidrug (co-)resistance to other drug classes such as macrolides, aminoglycosides, and fluoroquinolones [2,3]. This bacterium commonly colonizes the human nasal cavity and skin; however, when the skin barrier is compromised or host immunity is impaired, it can invade deep tissues, causing diseases ranging from skin and soft tissue infections to pneumonia, sepsis, and endocarditis [4].

The difficulty of treating MRSA is further compounded by its ability to form biofilms, bacterial communities encased in an extracellular polymeric matrix that acts as a physical barrier, impeding antibiotic penetration and shielding bacteria from immune clearance [5,6]. Although various anti-biofilm strategies have been explored, their clinical translation is often limited by high cost or toxicity [7,8]. Thus, the development of novel agents capable of eradicating both planktonic and biofilm-associated MRSA is urgently needed.

This challenge is particularly pronounced in diabetic patients, in whom MRSA infection is a major cause of chronic non-healing wounds such as diabetic foot ulcers [9,10]. Hyperglycemia-induced immune dysfunction, impaired neutrophil activity, and tissue hypoxia render diabetic wounds highly susceptible to bacterial colonization. Persistent infection releases cell-wall components and virulence factors, such as lipoteichoic acid, peptidoglycan fragments, and secreted toxins, which overactivate inflammatory responses and create a vicious cycle of “infection-inflammation-impaired healing” [11,12]. Furthermore, first-line anti-MRSA drugs such as vancomycin and linezolid are associated with severe adverse effects, including nephrotoxicity and myelosuppression, limiting their long-term use in diabetic wound management [13]. This clinical scenario therefore favors a safe, multifunctional agent that integrates antibacterial activity with pro-healing action.

**Figure 1:**
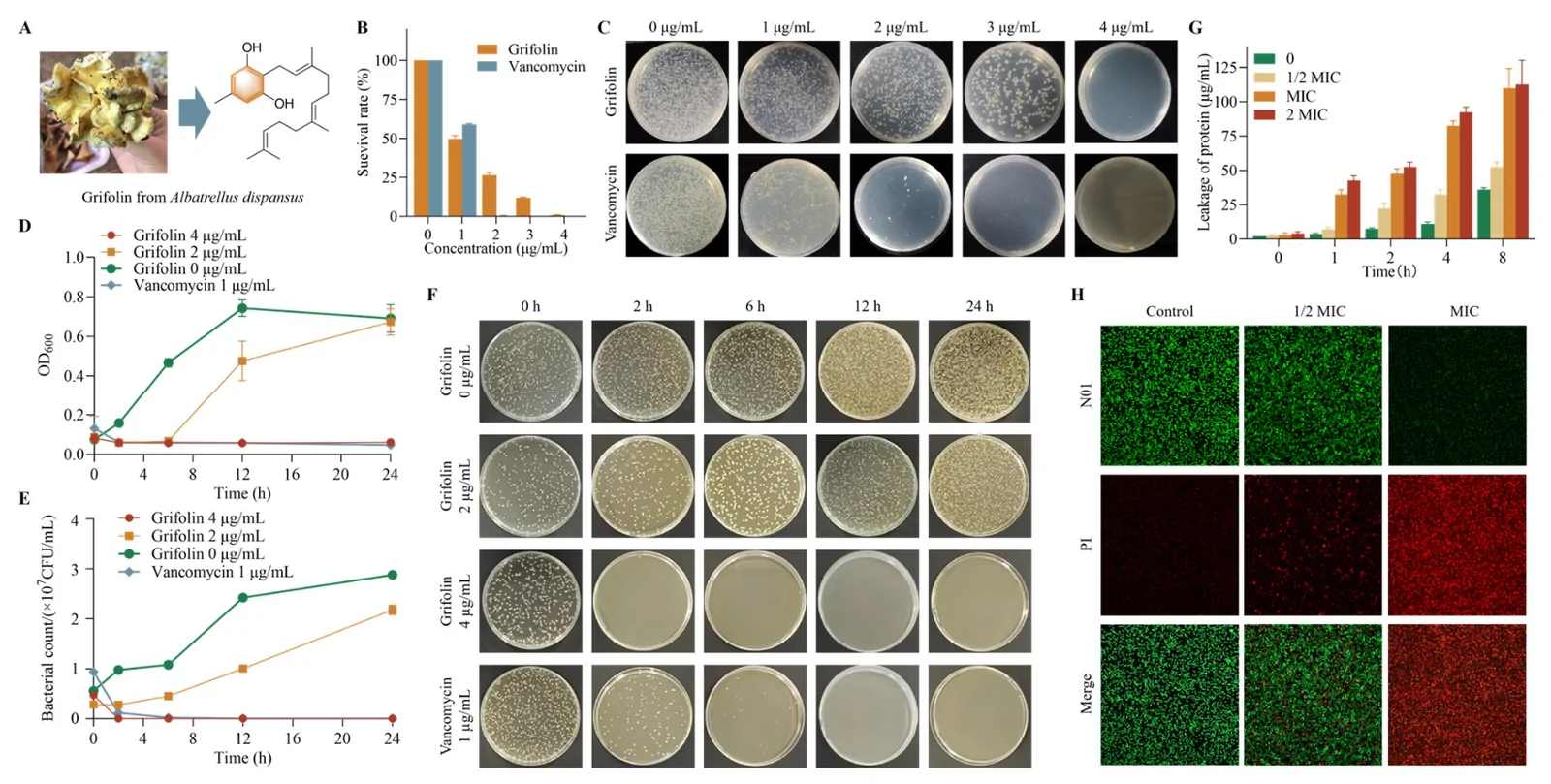
In vitro antibacterial activity of grifolin against planktonic MRSA. (**A**) Chemical structure of grifolin isolated from *Albatrellus dispansus*. (**B**,**C**) MRSA survival rates (**B**) and representative colony images (**C**) after treatment with grifolin or vancomycin. (**D**) Growth curves of MRSA within 24 h, as determined by OD_600_. (**E**,**F**) Time-kill kinetics (**E**) and representative colony images (**F**) of MRSA at different time points after treatment with grifolin or vancomycin, based on viable CFU counts. (**G**) Protein leakage from MRSA at different time points. (**H**) Representative confocal laser scanning microscopy images of MRSA treated without or with grifolin at 1/2 MIC and MIC, stained with SYTO 9 (live bacteria, green) and PI (dead bacteria, red). Data are expressed as mean ± SD (*n* = 3).

Grifolin, a phenolic compound we previously isolated from the edible fungus *Albatrellus dispansus* (Figure 1A), has attracted considerable attention due to its natural origin and diverse bioactivities [14]. It disrupts the bacterial cell envelope, suppresses pro-inflammatory mediators, and scavenges free radicals [15,16,17]. These multifaceted properties position grifolin as a promising candidate for infected diabetic wounds, which involve concurrent infection, inflammation and oxidative stress. However, its efficacy against MRSA, especially against mature biofilms, its in vivo therapeutic potential and its underlying molecular mechanisms remain unclear. Herein, we evaluated grifolin against planktonic and biofilm-embedded MRSA; integrated transcriptomic and proteomic profiling with qRT-PCR to explore the associated targets and pathways; and assessed its therapeutic performance in a murine MRSA-infected diabetic wound model.

## 2. Materials and Methods

### 2.1. Bacterial Strains and Reagents

The bacterial strains used in this study, including MRSA (ATCC 43300), MRSA (USA300), *Staphylococcus aureus* (*S. aureus*, ATCC 25923), and *Escherichia coli* (*E. coli*, ATCC 25922), were provided by Kunming Institute of Botany, Chinese Academy of Sciences (Kunming, China). Unless otherwise specified, MRSA refers to MRSA ATCC 43300. Vancomycin was purchased from Macklin company (Shanghai, China). Oxacillin, ampicillin, cefotaxime and cefoxitin were purchased from Aladdin (Shanghai, China). Streptozotocin was purchased from Biosharp (Beijing, China).

Grifolin was isolated from the fruiting body of *Albatrellus dispansus* by our group, and its structure was elucidated using spectroscopic methods [18]. Grifolin: yellow oil; C_22_H_32_O_2_; ^1^H NMR (600 MHz, methanol-*d4*) *δ*: 6.12 (2H, s, H-4 and H-6), 5.23 (1H, t, *J* = 7.1 Hz, H-2′), 5.08 (1H, t, *J* = 7.6 Hz, H-6′), 5.07 (H, t, *J* = 7.6 Hz, H-10′), 3.24 (2H, d, *J* = 7.1 Hz, H2-1′), 2.13 (3H, s, H3-7), 2.06 (2H, td, *J* = 7.8, 7.6 Hz, H2-9′), 2.01 (2H, td, *J* = 7.1, 7.6 Hz, H2-5′), 1.95 (2H, t, *J* = 7.8 Hz, H2-8′), 1.91 (2H, t, *J* = 7.1 Hz, H2-4′), 1.75 (3H, s, H3-15′), 1.65 (3H, s, H3-12′), 1.58 (3H, s, H3-13′), 1.56 (3H, s, H3-14′); ^13^C NMR (150 MHz, methanol-*d4*) *δ*: 156.9 (s, C-1 and C-3), 137.1 (s, C-5), 135.7 (s, C-7′), 134.4 (s, C-3′), 131.9 (s, C-11′), 125.6 (d, C-10′), 125.5 (d, C-6′), 125.1 (d, C-2′), 113.3 (s, C-2), 108.4 (d, C-4), 41.0 (t, C-8′), 40.8 (t, C-4′), 27.8 (t, C-5′), 27.6 (t, C-9′), 25.9 (q, C-12′), 22.9 (t, C-1′), 21.3 (q, C-7), 17.7 (q, C-13′), 16.2 (q, C-15′), 16.1 (q, C-14′).

### 2.2. Minimum Inhibitory Concentration (MIC) and Minimum Bactericidal Concentration (MBC)

The MIC of grifolin against planktonic bacteria was determined using the broth microdilution method according to CLSI guidelines [19]. Specifically, bacterial suspension of MRSA, USA300, *S. aureus* and *E. coli* were prepared by diluting to 5 × 10^5^ CFU/mL in tryptic soy broth (TSB), and 100 µL aliquots were dispensed into a 96-well plate. Grifolin was serially diluted and added to each well to achieve final concentration gradients. For MRSA, the concentrations tested were 1, 2, 3, and 4 μg/mL; for USA300, *S. aureus* and *E. coli*, the concentrations ranged from 0 to 32 μg/mL (0, 1, 2, 4, 8, 16, and 32 μg/mL). PBS-treated and vancomycin-treated bacterial suspensions served as the negative and positive control, respectively. The plate was incubated at 37 °C for 24 h. The MIC was defined as the lowest concentration of grifolin that inhibited visible growth (i.e., no observable turbidity).

The MBC was determined based on the MIC experiment, wherein 100 µL of the bacterial suspension from each well was evenly spread on TSA agar plates and incubated overnight at 37 °C. The MBC was defined as the lowest concentration at which the number of colonies was less than 0.1% of that in the negative control.

### 2.3. Time-Killing Kinetic Assay

The time-killing kinetic assay was performed as previously described with minor modifications [20]. MRSA was inoculated into 96-well plates at a density of 5 × 10^5^ CFU/mL and incubated at 37 °C. In the initial experiment, grifolin was added to the bacterial suspension at final concentrations of 0, 1, 3, and 4 μg/mL, and the optical density at 600 nm (OD_600_) was measured using a microplate reader (Tecan Spark 10M, Männedorf, Switzerland) at multiple time points (0, 2, 4, 10, 12, 18, and 24 h) to monitor bacterial growth. After the 24 h incubation, MTT was added to each well, followed by an additional 4 h incubation. The resulting formazan crystals were dissolved in DMSO, and the absorbance was measured at 570 nm. MRSA viability was calculated using the following formula: MRSA viability (%) = [(OD_sample_ − OD_blank_)/(OD_PBS_ − OD_blank_)] × 100%.

In a subsequent validation experiment, grifolin was tested at final concentrations of 0, 2, and 4 μg/mL, with vancomycin (1 μg/mL) included as a positive control. The OD_600_ was measured at 0, 2, 6, 12, and 24 h. Simultaneously, aliquots of each bacterial suspension were serially diluted, plated onto TSA agar plates, and incubated overnight at 37 °C. The colonies were then photographed and counted to determine the viable bacterial count (CFU/mL), and the time-killing curves were plotted based on the CFU counts over time.

### 2.4. Antibacterial Synergistic Effect of Grifolin Combined with Antibiotics on MRSA

The combined effects of grifolin and five conventional antibiotics (oxacillin, ampicillin, cefotaxime, cefoxitin, and vancomycin) against MRSA were evaluated by the checkerboard broth microdilution method [21,22]. The MIC of each antibiotic was first determined when the agents were used alone, and their MICs in combination were then determined under the same conditions. Specifically, in each 96-well plate, grifolin was serially diluted two-fold along the horizontal axis (columns) to establish a final concentration gradient ranging from 1 MIC to 1/256 MIC. Simultaneously, each antibiotic was serially diluted two-fold along the vertical direction (rows) to give final concentrations ranging from 1 MIC to 1/32 MIC. Thus, the antibiotic concentration was constant within each row and the grifolin concentration constant within each column. Sterility controls (sterile medium only) and growth controls (bacteria without either agent) were included on every plate for comparison. The plates were incubated at 37 °C for 24 h and the OD_600_ values were measured.

Wells whose OD_600_ remained at the baseline level of the sterility controls (approximately 0.05–0.08) were classified as non-turbid (no detectable bacterial growth), whereas wells with an OD_600_ above this level were classified as positive for growth. For every non-turbid well lying along the turbidity/non-turbidity interface, the fractional inhibitory concentration (FIC) of each agent was calculated as the ratio of its MIC in combination to its MIC alone: FIC_A_ = MIC_A(combine)_/MIC_A(alone)_, FIC_B_ = MIC_B (combine)_/MIC_B(alone)_, where A denotes grifolin and B denotes the other tested antibiotic. The FIC index (FICI) for the same well was calculated as FICI = FIC_A_ + FIC_B_. FICI values were determined for all non-turbid wells along the growth/no-growth interface, and the lowest FICI (ΣFICmin) was used to characterize each interaction. Interactions were classified using the following commonly adopted thresholds, chosen to maintain comparability with the published literature: synergistic when FICI ≤ 0.5, additive when 0.5 < FICI ≤ 1.0, indifferent when 1.0 < FICI ≤ 4, and antagonistic when FICI > 4.0 [23,24].

### 2.5. MRSA Biofilm Scavenging

The efficacy of grifolin in eradicating mature MRSA biofilms was evaluated using both crystal violet staining and resazurin-based fluorescence assays, as previously described with minor modifications [25,26]. Briefly, MRSA suspensions were inoculated into 96-well plates (black-walled opaque plates for fluorescence assay) and incubated at 37 °C for 48 h to allow the formation of mature biofilms. After incubation, the culture supernatant was carefully aspirated, and floating planktonic bacteria were removed by gentle PBS washing. TSB containing grifolin at various concentrations (crystal violet assay: 0, 4, 5, 6, 7, and 8 μg/mL; resazurin assay: 1/2 MIC, 1 MIC, 2 MIC, and 3 MIC) or vancomycin (positive control, same concentration gradient) was added. The plates were then incubated at 37 °C for another 24 h, followed by gentle PBS washing.

For crystal violet staining, the remaining biofilms were fixed with methanol for 30 min at room temperature, stained with a 0.1% (*w*/*v*) crystal violet for 30 min, and washed with PBS. Then, 33% glacial acetic acid was added to dissolve the purple precipitate. The OD_600_ was measured to quantify the biofilm biomass. The biofilm clearance rate was calculated using the following formula: Biofilm clearance rate (%) = (1 − OD_Sample_/OD_PBS_) × 100%.

For the resazurin-based fluorescence assay, fresh TSB containing resazurin reagent (BacTiter-Fluo™ Microbial Cell Viability Assay Kit, Beyotime, Shanghai, China) was added to each well, and the plates were incubated at 37 °C for 30 min. The fluorescence intensity was measured using a microplate reader (Tecan Spark 10M, Männedorf, Switzerland) at an excitation wavelength of 560 nm and an emission wavelength of 600 nm. After the fluorescence assay, the remaining biofilms were harvested and resuspended in sterile PBS. The bacterial suspensions were serially diluted, plated onto TSA plates, and incubated at 37 °C for 24 h. The viable bacterial counts were determined as CFU per well.

### 2.6. Determination of Protein Leakage

Bacterial suspensions supplemented with grifolin at final concentrations of 0, 1/2 MIC, MIC and 2 MIC were incubated at 37 °C for intervals of 0, 1, 2, 4 and 8 h. Following incubation, the samples were centrifuged at 8000 rpm to pellet the bacteria [27]. The concentration of protein leakage in the supernatant was determined using the BCA Protein Assay Kit (Beyotime, Shanghai, China).

### 2.7. Live/Dead Staining

5 × 10^5^ CFU/mL of MRSA was incubated with grifolin at concentrations of 0, 1/2 MIC and MIC at 37 °C for 2 h. After harvested, the bacterial cells were stained with SYTO9 (N01) dye and propidium iodide (PI) dye at RT for 15 min in sequence. Subsequently, the cells were rinsed 2–3 times with PBS and visualized under confocal laser scanning microscopy (CLSM, Carl Zeiss LSM 900, Oberkochen, Germany). In addition, mature biofilms were formed following the aforementioned method and incubate with grifolin at concentrations of 0, 1 MIC and 2 MIC at 37 °C for 2 h. Upon live/dead staining, the three-dimensional images of the biofilms were acquired using CLSM in *Z*-axis mode [28].

### 2.8. Hemolytic Assay

Fresh red blood cells (RBCs) were isolated from rabbit blood. Then, 100, 50, 25, 12.5, 6.25, 3.125, 1.56, 0.78 and 0.39 µg/mL of grifolin were added to the 96-well plates containing an 8% (*v*/*v*) RBC suspension. The mixtures were subsequently incubated at 37 °C for 2 h and then centrifuged at 3000 rpm for 5 min. The supernatant was carefully transferred to a microplate, and absorbance was measured at 572 nm. Deionized water (DI) served as the positive control, while PBS served as the negative control [29]. The percentage of hemolysis was calculated using the following equation: Hemolysis (%) = [(OD_Sample_ − OD_PBS_)/(OD_DI_ − OD_PBS_)] × 100%.

### 2.9. Transcriptome and Proteome Analysis

MRSA cultures in the logarithmic growth phase were divided into two groups: the grifolin-treated group (G group), exposed to grifolin at 1.5 μg/mL (1/2 MIC), and the MRSA control group (M group), treated with an equal volume of PBS. Three independent biological replicates were performed for each group (G1–G3 and M1–M3). After 3 h of co-incubation at 37 °C, total RNA and proteins were extracted for transcriptomic and proteomic profiling, respectively. Transcriptomic analysis was performed using the Illumina high-throughput sequencing platform, while proteomic analysis was conducted using 4D-DIA-based quantitative mass spectrometry. Detailed experimental procedures are provided in the Supplementary Materials. To investigate the metabolic pathways and biological functions associated with the differentially expressed genes (DEGs) and differentially expressed proteins (DEPs), Gene Ontology (GO), Kyoto Encyclopedia of Genes and Genomes (KEGG) pathway enrichment, and gene set enrichment analyses (GSEA) were performed on the Majorbio Cloud Platform [30]. The raw RNA sequencing data have been deposited in the NCBI Sequence Read Archive (SRA) under accession number PRJNA1458645. The mass spectrometry proteomics data have been deposited to the ProteomeXchange Consortium Via the PRIDE partner repository with the dataset identifier PXD086612.

### 2.10. Quantitative Reverse Transcriptional PCR (qRT-PCR)

To justify the use of 16S rRNA as the reference gene for qRT-PCR normalization, the expression stability of five candidate reference genes (16S rRNA, *gapA*, *gyrB*, *rpoB*, and *tuf*) under grifolin treatment was evaluated using the geNorm algorithm [31]. The stability measure M reflects the average pairwise variation in each candidate gene’s expression ratio with the other candidates across samples; genes with higher M values are sequentially removed until the two most stable genes remain, yielding the stability ranking. M < 0.5 is generally considered stable in relatively homogeneous sample sets. qRT-PCR was then employed to validate the RNA-Seq results through targeted gene expression analysis of *clfB*, *moeA*, *agrA*, *murQ*, *vwb*, *mecA*, *icaD*, *rplN*, *dltB* and *infC*.

Specifically, total RNA was extracted from both the grifolin-treated and control groups, followed by reverse transcription using the ABScript Neo RT Master Mix for qPCR with gDNA Remover (Abclonal, Wuhan, China) as per the manufacturer’s instructions. Gene expression was analyzed on a real-time PCR instrument (CFX system, Bio-rad, Hercules, CA, USA) using the BrightCycle Universal SYBR Green qPCR Mix with UDG (Abclonal, Wuhan, China). Thermal cycling conditions commenced with an initial denaturation at 95 °C for 3 min, followed by 40 cycles consisting of denaturation at 95 °C for 5 s and extension at 60 °C for 30 s, and concluded with melting curve analysis. The reference gene was 16S rRNA, and relative quantification of gene expression was determined using the 2^^−ΔΔCT^ method. The primer sequences are detailed in Supplementary Table S3.

### 2.11. Infected Diabetic Wound Healing in Mice

Animal experiments were approved by Ethics Committee of South-Central Minzu University (Approval No. SYXM (Hubei) 2021-0089, Protocol No. 2023-SCUEC-AEC-033), and were conducted in strict compliance with the committee’s guidelines. Kunming mice (18–20 g, 6–8 weeks old, male) were obtained from Hunan Sja Laboratory Animal Co., Ltd. (License No. SCXK (Xiang) 2019-0004). Following a 7-day period of routine adaptive feeding, mice with normal physiological status were subjected to intraperitoneal injection of streptozotocin (STZ, 120 mg/kg) after an overnight fast [32,33]. Fasting blood glucose levels were measured over the subsequent week, and the diabetic model was considered successfully established when blood glucose concentrations reached or exceeded 16.7 mmol/L. Subsequently, a full-thickness skin wound with an 8 mm diameter was created on the right dorsal region of each mouse. MRSA bacterial suspension (1 × 10^6^ CFU/mL, 100 µL) was inoculated into the wound [34]. After 24 h, evident pus exudation was observed in the wounds, confirming the successful establishment of the diabetic wound infection model.

Based on the In Vitro effective concentrations and a preliminary tolerability assessment, we randomly assigned the infected mice to four groups (*n* = 8 per group) using a computer-generated random number table: the model group, grifolin low-dose (GL, 5 mg/kg) group, the grifolin high-dose (GH, 10 mg/kg) group and vancomycin (10 mg/kg) group. Grifolin was dissolved in DMSO, supplemented with PEG 400 as a co-solvent, and diluted with PBS to the required working concentration; the final concentrations of DMSO and PEG 400 in all formulations were below 1% and 10%, respectively. Vancomycin was dissolved directly in PBS. On day 1 after infection, a single dose of 100 µL of the corresponding formulation was evenly applied to the wound surface, and the wound was covered with a 3M transparent film dressing to prevent contamination and ensure sustained drug exposure. The model group received an equal volume of vehicle (PBS containing the same concentrations of DMSO and PEG 400). Group allocation was performed by an investigator who was not involved in outcome assessment. Wound-area measurement, histological evaluation, immunofluorescence imaging, and CFU counting were conducted by blinded investigators who were unaware of the group assignments. The wound status and body weight of the mice were recorded on days 0, 3, 7, 10 and 13 post-inoculation. The relative wound area was calculated as follows: Relative wound area (%) = A_time_/A_initial_ × 100%, where A_initial_ denotes the initial wound area (on day 0), and A_time_ denotes the wound area at various time points.

On day 7, three mice per group were euthanized, and wound tissues were collected for hematoxylin and eosin (H&E) staining, Masson’s trichrome staining, immunofluorescence analysis and bacterial load analysis. For CFU determination, wound tissues were homogenized in sterile normal saline, serially diluted, and cultured on TSA plates for 24 h. The remaining five mice per group were monitored until day 13 and completed the full wound-closure observation. On day 13, three mice were randomly selected from the five remaining mice in each group for additional histological evaluation and bacterial load analysis. At the end of the experiment, all mice were euthanized, and major organs (heart, liver, spleen, lung, and kidney) were harvested and subjected to H&E staining to evaluate systemic toxicity.

### 2.12. Statistical Analysis

GraphPad Prism 8.0 was used for statistical analysis. Unless otherwise specified, all experiments were performed in three independent biological replicates. Technical replicate wells within each experiment were averaged before statistical analysis. Single-endpoint comparisons were analyzed by one-way analysis of variance (ANOVA) with Tukey’s test, while the longitudinal wound-closure data were analyzed by two-way repeated-measures ANOVA with treatment and time as factors, followed by Sidak’s post hoc test. *p* < 0.05 was regarded as significance.

## 3. Results

### 3.1. Bactericidal Activity of Grifolin Against Planktonic MRSA

The antibacterial activity of grifolin against planktonic MRSA was first evaluated. Grifolin exhibited potent activity with an MIC of 3 μg/mL and an MBC of 4 μg/mL, indicating its bactericidal nature. The survival rate of MRSA decreased in a concentration-dependent manner following grifolin treatment, with almost no visible colony at 4 μg/mL, and its efficacy was comparable to that of vancomycin (Figure 1B,C). Time-killing kinetics revealed that grifolin exerted a rapid and sustained bactericidal effect against MRSA over 24 h. At its MIC (3 μg/mL), grifolin markedly reduced the viable bacterial count to an extent comparable to that of 1 μg/mL vancomycin (Supplementary Figure S1A). In a subsequent validation experiment, grifolin at 2 μg/mL markedly suppressed bacterial growth without complete eradication, whereas grifolin at 4 μg/mL rapidly reduced viable bacteria below the detection limit, which was maintained throughout the 24 h observation period (Figure 1D–F). Consistently, the MTT assay confirmed concentration-dependent inhibition of bacterial metabolic activity, with viability reduced to nearly zero at the MBC (Supplementary Figure S1B).

Next, we examined changes in bacterial membrane integrity upon grifolin treatment. A dose- and time-dependent increase in extracellular protein concentration was detected by the BCA assay (Figure 1G), suggesting substantial leakage of cytoplasmic contents. This membrane damage was directly visualized by live/dead staining coupled with confocal microscopy (Figure 1H). Untreated MRSA cells displayed predominantly green fluorescence (SYTO 9, intact membranes), while treatment with grifolin at 1/2 MIC and MIC resulted in a marked increase in red fluorescence due to propidium iodide (PI) uptake, confirming widespread membrane damage and cell death. Collectively, these results establish grifolin as a potent bactericidal agent against planktonic MRSA, whose activity is associated with disruption of bacterial cytoplasmic membrane integrity.

To further evaluate the antibacterial spectrum of grifolin, its activity was examined against *S. aureus* (ATCC 25923), a standard methicillin-susceptible quality-control strain; the hypervirulent community-associated MRSA strain USA300; and a Gram-negative reference strain *E. coli* (ATCC 25922). Grifolin exhibited an MIC and MBC of 2 μg/mL against both *S. aureus* and USA300, indicating even stronger bactericidal activity than against MRSA ATCC 43300. Both OD_600_ and viable CFU counts decreased in a concentration-dependent manner, with almost no visible colonies at 2 μg/mL and above, and the bactericidal effect was comparable to that of 1 μg/mL vancomycin (Supplementary Figure S2). In contrast, grifolin showed no detectable inhibitory activity against *E. coli* at concentrations up to 32 μg/mL, indicating a selective action against Gram-positive bacteria. The MIC and MBC values for all tested strains are summarized in Supplementary Table S1.

### 3.2. Eradication of Mature MRSA Biofilms by Grifolin

**Figure 2:**
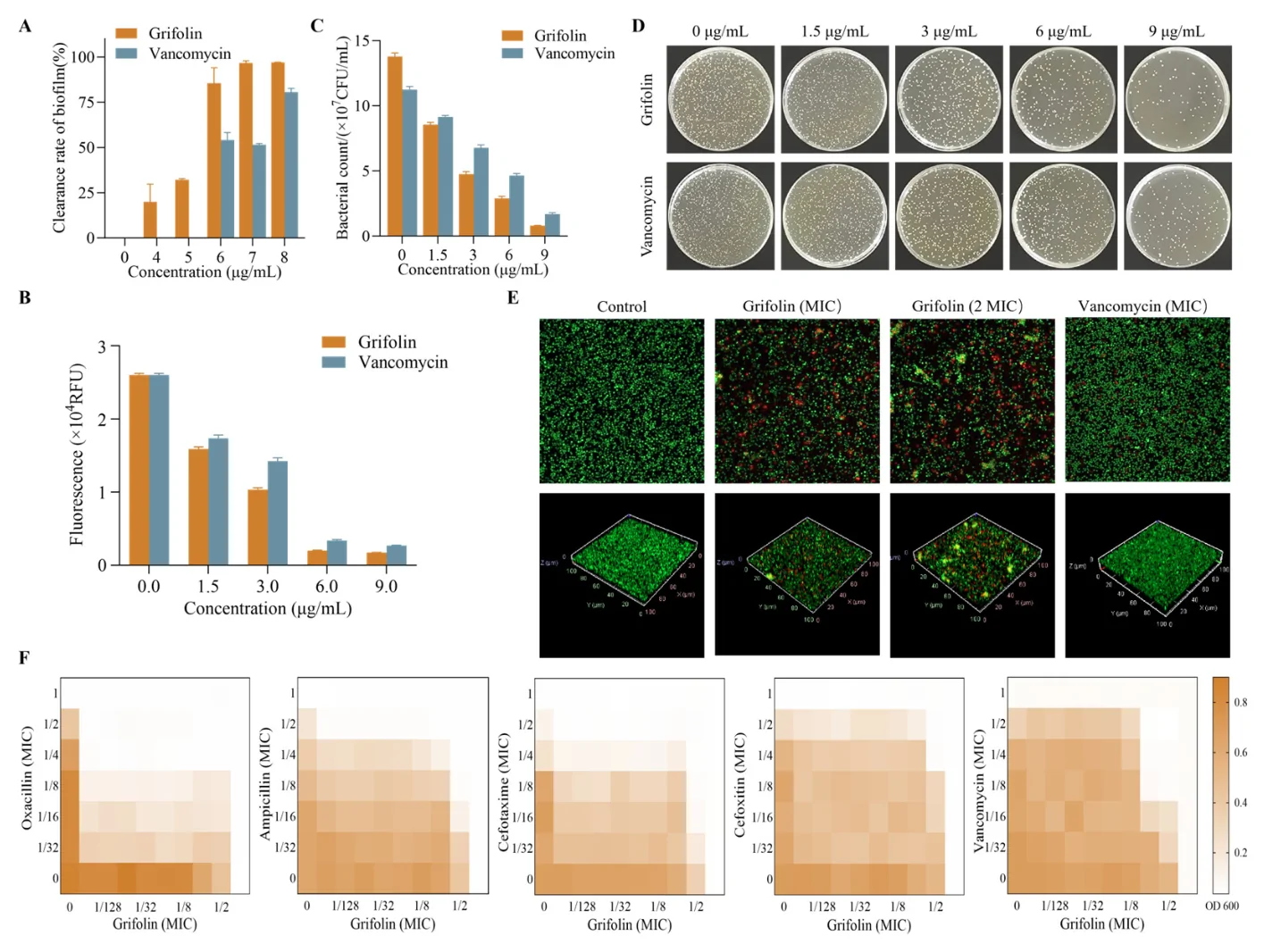
In vitro anti-MRSA biofilm and synergistic activities of grifolin. (**A**) Clearance of mature MRSA biofilms by grifolin and vancomycin, determined by crystal violet staining. (**B**) Viability of MRSA biofilms treated with grifolin or vancomycin, determined by resazurin-based fluorescence assay. (**C**,**D**) Viable CFU counts (**C**) and representative colony images (**D**) of MRSA biofilms treated with grifolin or vancomycin. (**E**) Representative 2D planar and 3D stereoscopic CLSM images of mature MRSA biofilms following treatment with grifolin (MIC, 2MIC) or vancomycin (MIC), stained with SYTO 9 (live bacteria, green) and PI (dead bacteria, red). (**F**) Antibacterial effects of grifolin in combination with conventional antibiotics (oxacillin, ampicillin, cefotaxime, cefoxitin, vancomycin) against planktonic MRSA, as assessed by checkerboard assay. Data are expressed as mean ± SD (*n* = 3).

Given that biofilms are central to chronic infections, we evaluated grifolin’s activity against pre-formed MRSA biofilms [35]. Crystal violet quantification showed that grifolin dose-dependently reduced mature biofilm biomass, with significant eradication observed at 2 MIC (Figure 2A). Notably, at equivalent multiples of their respective MICs, grifolin exhibited biofilm-clearing activity that compared favorably with vancomycin. To determine whether this loss of biomass reflected genuine killing rather than simple dispersal, biofilm metabolic viability was examined using a resazurin-based fluorescence assay (Figure 2B). Grifolin produced a dose-dependent decline in fluorescence, indicating a progressive reduction in metabolically active, viable cells, and this effect was consistently greater than that achieved by vancomycin at matched MIC multiples. For a direct, culture-based confirmation, the resazurin assay wells were then serially diluted and plated to enumerate viable CFU (Figure 2C, D). Grifolin reduced the number of recoverable culturable cells in a dose-dependent manner: at 9 μg/mL (3 MIC), the surviving fraction was only 5.8% of the untreated control, whereas vancomycin at the same concentration was less effective, with 15.2% of the cells still viable. These CFU data were fully consistent with the fluorescence and crystal violet biomass measurements, confirming that grifolin exerts true bactericidal action against biofilm-embedded MRSA rather than merely detaching the biofilm matrix.

This anti-biofilm efficacy was visually confirmed by 3D confocal imaging of live/dead-stained biofilms (Figure 2E). Untreated, mature biofilms presented as dense, architecturally complex communities with dominant green fluorescence, indicating high viability. In stark contrast, treatment with grifolin at its MIC and 2 MIC drastically reduced the biofilm biovolume, disrupted the 3D structure, and replaced green fluorescence with intense red signals, indicating a high proportion of dead cells. Vancomycin at its MIC, however, showed limited disruption, with biofilms retaining considerable green fluorescence and structural integrity. These results unequivocally establish that grifolin, unlike vancomycin, retains potent bactericidal activity against the biofilm-embedded, recalcitrant form of MRSA.

### 3.3. Effect of Grifolin in Combination with Antibiotics Against MRSA

To explore the potential of combining grifolin with conventional antibiotics, the combined antibacterial activity of grifolin paired with five clinically relevant antibiotics, oxacillin, ampicillin, cefotaxime, cefoxitin and vancomycin, against MRSA was assessed [36]. The FICI was then employed to evaluate the nature of these combined effects. As shown in the checkerboard heatmap in Figure 2F and Supplementary Table S2, grifolin acted synergistically with two of the five antibiotics. The grifolin-oxacillin combination exhibited the most pronounced interaction, with an FICI of 0.2539, and the grifolin-vancomycin combination also showed a synergistic effect, with an FICI of 0.375. The remaining three combinations were classified as additive: the FICIs for ampicillin and cefotaxime were each 0.5039, and that for cefoxitin was 0.75. With respect to oxacillin and vancomycin, even at their sub-inhibitory concentrations, combination with grifolin markedly reduced the OD_600_ of the culture system, indicating strong suppression of bacterial growth, and this effect showed clear concentration dependence, validating the two synergistic interactions [37]. For ampicillin, cefotaxime and cefoxitin, enhanced antibacterial activity was observed only when they were present at relatively high concentrations, which is consistent with the characteristics of additive effects. These findings provide experimental evidence for combination regimens using grifolin as an antibiotic-sensitizing agent, including the reversal of resistance to the β-lactam oxacillin and the potentiation of the glycopeptide vancomycin.

### 3.4. Antibacterial Mechanism of Grifolin Revealed by Multi-Omics

**Figure 3:**
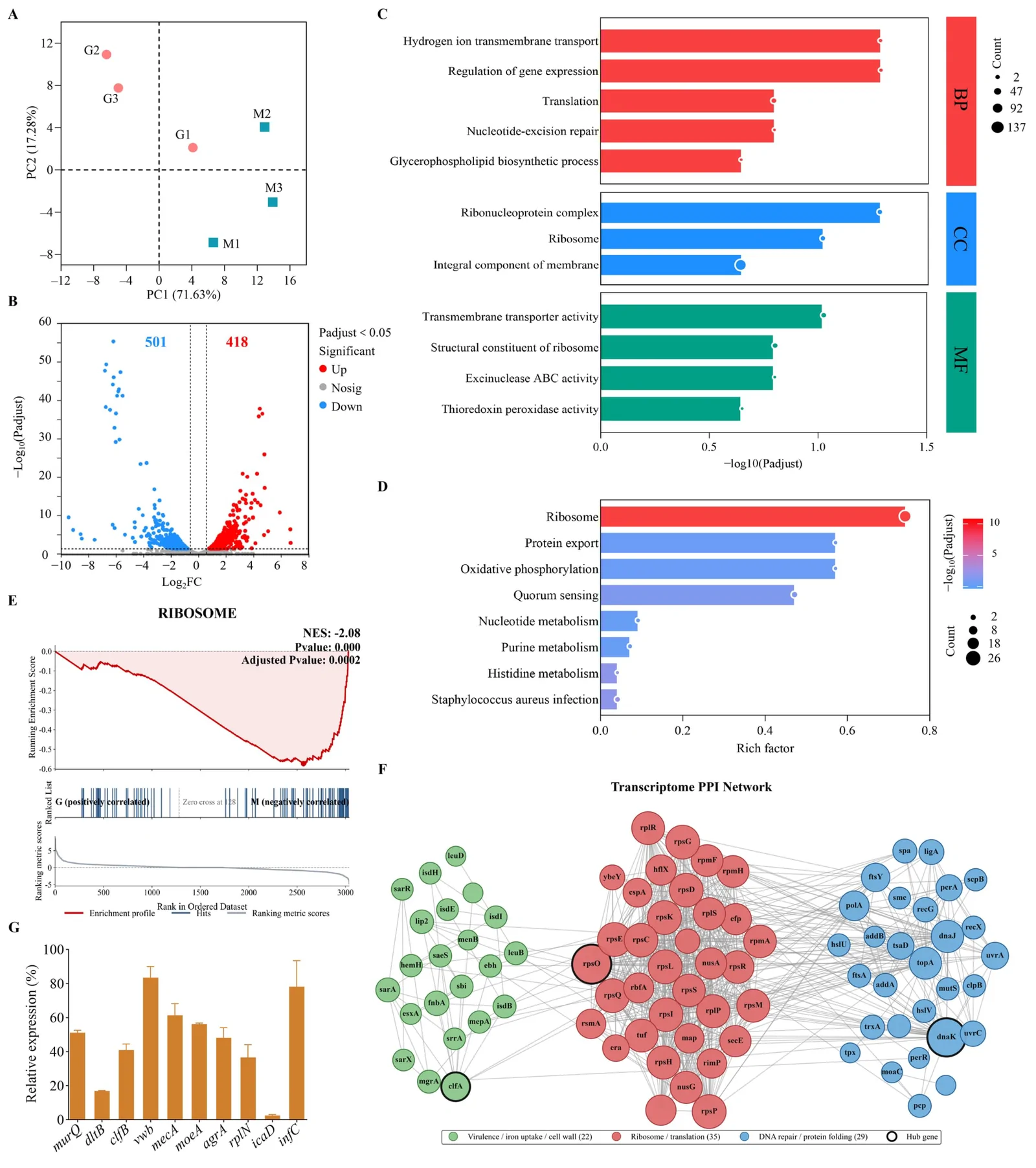
Transcriptomic profiling of MRSA upon grifolin treatment. (**A**) PCA of grifolin-treated (G1–G3) and untreated MRSA control (M1–M3) replicates. (**B**) Volcano plot of DEGs following grifolin treatment, where red and blue dots denote significantly up-regulated (418) and down-regulated (501) genes (Padjust < 0.05). (**C**) GO enrichment analysis across biological process (BP), cellular component (CC) and molecular function (MF) categories. (**D**) KEGG pathway enrichment analysis of DEGs ranked by rich factor. (**E**) GSEA of the ribosome gene set. (**F**) PPI networks derived from the transcriptome data with nodes colored by module. (**G**) RT-qPCR validation of selected DEGs, presented as relative expression compared to the untreated control.

To systematically elucidate the mechanism of grifolin against MRSA, we integrated transcriptomic and proteomic profiling together with qRT-PCR validation, dissecting its antibacterial effects at the levels of gene transcription and protein expression. Principal component analysis (PCA) showed that the grifolin-treated (G1–G3) and untreated MRSA control (M1–M3) samples formed two clearly separated clusters, indicating that grifolin induced globally transcriptomic remodeling (Figure 3A). RNA-seq identified a total of 919 DEGs, comprising 501 downregulated and 418 upregulated genes, as displayed in the volcano plot (Figure 3B). GO analysis resolved the enriched terms into three ontologies (Figure 3C). In the biological process (BP) category, the DEGs were enriched in hydrogen ion transmembrane transport, regulation of gene expression, translation, nucleotide-excision repair and the glycerophospholipid biosynthetic process, indicating coordinated changes in gene expression, protein synthesis and the DNA damage response. The cellular component (CC) category was dominated by the ribonucleoprotein complex and ribosome, together with the integral component of the membrane, in line with the pervasive effects on translation and membrane architecture. In the molecular function (MF) category, the enriched terms comprised transmembrane transporter activity, the structural constituent of ribosomes, exonuclease ABC activity and thioredoxin peroxidase activity, reflecting altered membrane transport, ribosomal structure and antioxidant/DNA-repair enzyme functions. In agreement, KEGG enrichment analysis highlighted that the DEGs were most strongly enriched in the ribosome pathway, followed by protein export, oxidative phosphorylation and quorum sensing, together with nucleotide, purine and histidine metabolism and the *Staphylococcus aureus* infection pathway (Figure 3D). GSEA further supported that the ribosome (NES = −2.08, adjusted *p* = 0.0002) gene sets were significantly and negatively enriched following grifolin treatment (Figure 3E), confirming downregulation of translation program.

To further resolve the functional organization of these transcriptional changes at the network level, a protein–protein interaction (PPI) network was constructed and the three most prominent and mechanistically relevant modules were selected for display (Figure 3F). The largest module (35 nodes; hub rpsO) was dedicated to ribosome/translation and was densely populated with ribosomal proteins and translation factors. A second module (29 nodes; hub dnaK) grouped DNA-repair and protein-folding factors, whereas the third (22 nodes; hub clfA) assembled virulence, iron-uptake and cell-wall factors [38]. Numerous cross-module edges connected these three clusters, indicating that translation, genome maintenance and virulence/cell-envelope functions are tightly coupled and coordinately suppressed by grifolin.

To validate the RNA-seq results by qRT-PCR, we first confirmed the reliability of 16S rRNA as the internal reference gene under control and grifolin-treatment conditions (Supplementary Figure S3). Its mean Ct values were nearly identical between groups (8.2426 ± 0.0490 vs. 8.1864 ± 0.1471, ΔCt ≈ 0.056 cycles). GeNorm analysis of five candidate genes spanning distinct functional categories (16S rRNA, *gapA* for metabolism, *gyrB* for DNA replication, *rpoB* for transcription, and *tuf* for translation) showed all M values well below the 0.5 threshold, with 16S rRNA at approximately 0.18, confirming its stable expression under grifolin treatment (Supplementary Table S4). Using this validated reference gene, 10 key genes were selected for RT-qPCR analysis (Figure 3G), covering cell wall biosynthesis (*murQ*, *dltB*), adhesion (*clfB*), virulence (*vwb*), drug resistance (*mecA*), cofactor metabolism (*moeA*), quorum sensing (*agrA*), ribosomal protein synthesis (*rplN*), biofilm formation (*icaD*) and protein synthesis (*infC*). All showed downregulation consistent with the transcriptomic data. Specifically, downregulation of *murQ* and *dltB* directly verified impaired peptidoglycan and teichoic acid biosynthesis, providing a molecular basis for the observed cell wall/membrane damage [39,40]. Downregulation of *moeA*, which is required for molybdenum cofactor supply, pointed to perturbed cofactor-dependent metabolism [41], while reduced *rplN* and *infC* expression indicated impaired ribosome assembly and protein synthesis, thereby restricting bacterial growth and repair [42]. Decreased expression of *clfB*, *vwb* and *icaD* attenuated adhesion, virulence factor activity, and biofilm formation, weakening pathogenicity and environmental adaptability. Notably, the marked downregulation of *mecA* and *agrA* revealed two deeper mechanisms: reduced *mecA* may constitute a core basis for reversing resistance to β-lactams such as oxacillin, whereas reduced *agrA* disrupts the quorum-sensing network that coordinates group behavior, thereby fundamentally attenuating virulence [43].

**Figure 4:**
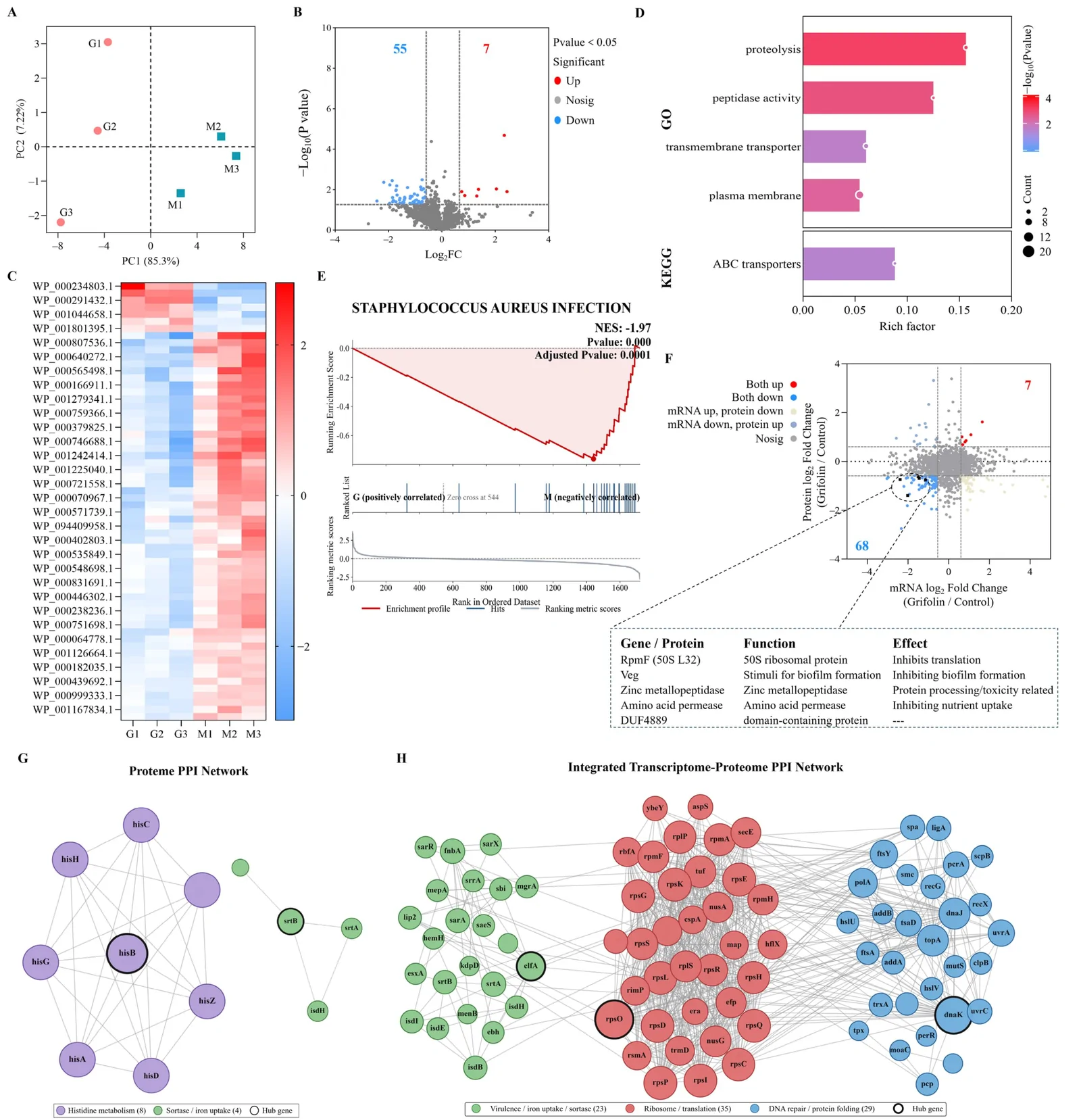
Proteomic profiling and integrated transcriptome-proteome analysis of MRSA upon grifolin treatment. (**A**) PCA of grifolin-treated (G1–G3) and untreated MRSA control (M1–M3) replicates. (**B**) Volcano plot of DEPs following grifolin treatment, where red and blue dots denote significantly up-regulated (7) and down-regulated (55) genes (*p* < 0.05). (**C**) Hierarchical clustering heatmap of DEPs; red and blue indicate high and low relative expression, respectively. (**D**) GO and KEGG pathway enrichment analysis of DEPs ranked by rich factor. (**E**) GSEA of the *Staphylococcus aureus* infection gene set. (**F**) Nine-quadrant scatter plot relating mRNA and protein Log_2_ fold changes, distinguishing concordant and discordant regulation, with representative genes/proteins and their functions listed below. (**G**,**H**) PPI networks derived from the proteome (**G**) and the integrated transcriptome-proteome (**H**) data, with nodes colored by module.

Complementing the transcriptomic data, label-free proteomics quantified the protein-level response to grifolin, from which 12,670 peptides were identified and assigned to 1775 distinct proteins. The Pearson correlation heatmap showed high pairwise correlations among all samples (r = 0.92–1.00), while the peptide-count distribution per protein reflected adequate identification depth; demonstrating good quantitative reproducibility and reliability for the subsequent analyses (Supplementary Figure S4). PCA clearly separated the grifolin-treated and control samples (Figure 4A), and 62 DEPs were identified, of which 55 were downregulated and 7 upregulated (Figure 4B). Hierarchical clustering of the DEPs clearly separated the grifolin-treated and control groups, with the DEPs showing consistent expression patterns within each group (Figure 4C). GO and KEGG enrichment analyses (Figure 4D) revealed that these proteins were concentrated in membrane-associated functions: the GO terms proteolysis, peptidase activity, transmembrane transporter and plasma membrane were enriched, together with the KEGG ABC transporters pathway, indicating perturbed protein degradation and membrane transport. GSEA further showed that the *Staphylococcus aureus* infection gene set was significantly negatively enriched (NES = −1.97, adjusted *p* = 0.0001), confirming coordinated suppression of virulence-related programs (Figure 4E).

Cross-omics comparison by the nine-quadrant analysis revealed concordant expression trends, with 7 genes consistently upregulated and 68 genes consistently downregulated at both the mRNA and protein levels (Figure 4F). Among the concordantly downregulated genes, five representative proteins with *p* < 0.05 are highlighted: RpmF (50S ribosomal protein L32) is ribosomal protein whose reduction inhibits translation; Veg, a biofilm-formation stimulus, was suppressed to inhibit biofilm formation; the zinc metallopeptidase was reduced to affect protein processing and toxicity; and the amino acid permease was downregulated to limit nutrient uptake [44]. These concordant changes indicate that the transcriptional repression was reflected at the protein level, jointly impairing translation, biofilm formation, protein processing and nutrient acquisition. Moreover, the proteome PPI network resolved two core modules (Figure 4G): the histidine metabolism module (hub hisB) and the sortase/iron-uptake module (hub srtB). The integrated transcriptome-proteome PPI network construction and core module extraction (Figure 4H) further merged these proteins with the transcriptomic virulence, ribosome/translation and DNA-repair/protein-folding modules [45].

Collectively, the transcriptomic and proteomic results converge on a coherent, multi-target mechanism: grifolin coordinately suppresses ribosome/translation, cell-wall and membrane integrity, energy and nutrient metabolism, quorum sensing and virulence, providing a mechanistic basis for its anti-MRSA activity.

### 3.5. Therapeutic Efficacy of Grifolin in MRSA-Infected Diabetic Wound

**Figure 5:**
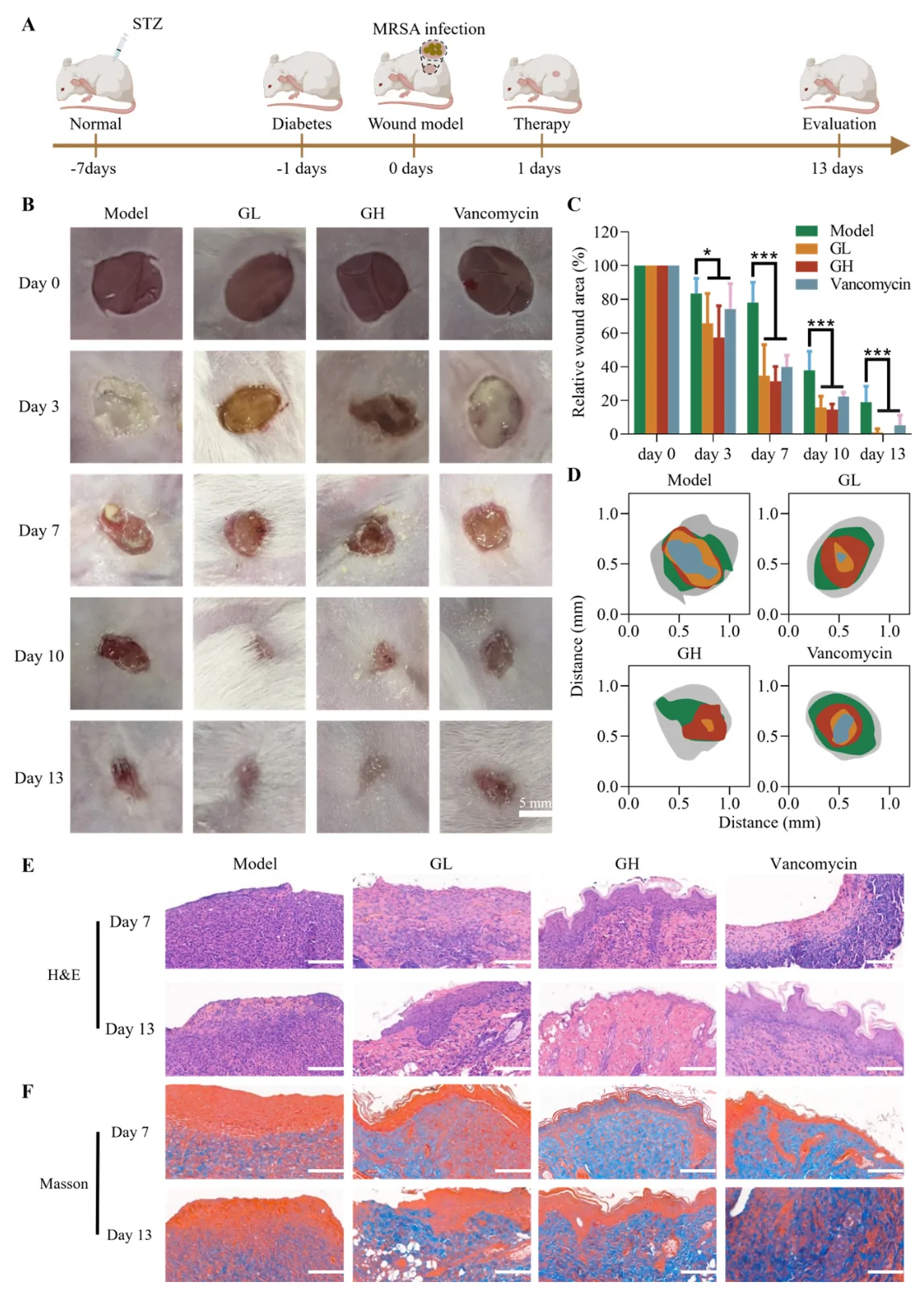
In vivo therapeutic evaluation of grifolin in a murine model of MRSA-infected diabetic wounds. (**A**) Schematic timeline of the animal experiment, including diabetes induction, MRSA-infected wound establishment, treatment and evaluation. (**B**) Representative photographs of MRSA-infected diabetic wounds on days 0, 3, 7, 10 and 13. Scale bar = 5 mm. (**C**) Relative wound area (wound closure) following different treatments (*n* = 5). * *p* < 0.05 and *** *p* < 0.001, compared with the model group. (**D**) Digital tracings of wound areas on days 0, 3, 7, 10 and 13. (**E**,**F**) Representative H&E (**E**) and Masson (**F**) stained wound tissue sections on days 7 and 13. Scale bar = 100 μm.

To evaluate the in vivo antibacterial efficacy of grifolin, a murine model of MRSA-infected diabetic wounds was established (Figure 5A) [46]. As shown in the macroscopic wound images, grifolin treatment significantly facilitated wound healing and tissue regeneration (Figure 5B). Dynamic monitoring revealed that grifolin treatment could accelerate wound closure in a dose-dependent manner (Figure 5C,D). On day 3, the wound in the high-dose grifolin (GH) group had markedly contracted with no pus leakage, whereas the model group and the vancomycin group still exhibited obvious signs of infection. By day 13, the wounds in the high-dose grifolin group had almost fully re-epithelialized, with healing comparable to that in the vancomycin group.

Histological analysis (H&E and Masson’s trichrome staining) further confirmed these findings (Figure 5E,F). Grifolin, particularly at the high dose, effectively promoted tissue reconstruction by reducing inflammatory cell infiltration, increasing collagen deposition density, and accelerating the formation of a compact, well-structured neo-epidermis. These results demonstrate that grifolin not only accelerates wound coverage but also improves the quality of tissue repair, supporting its therapeutic potential for diabetic wound infection.

**Figure 6:**
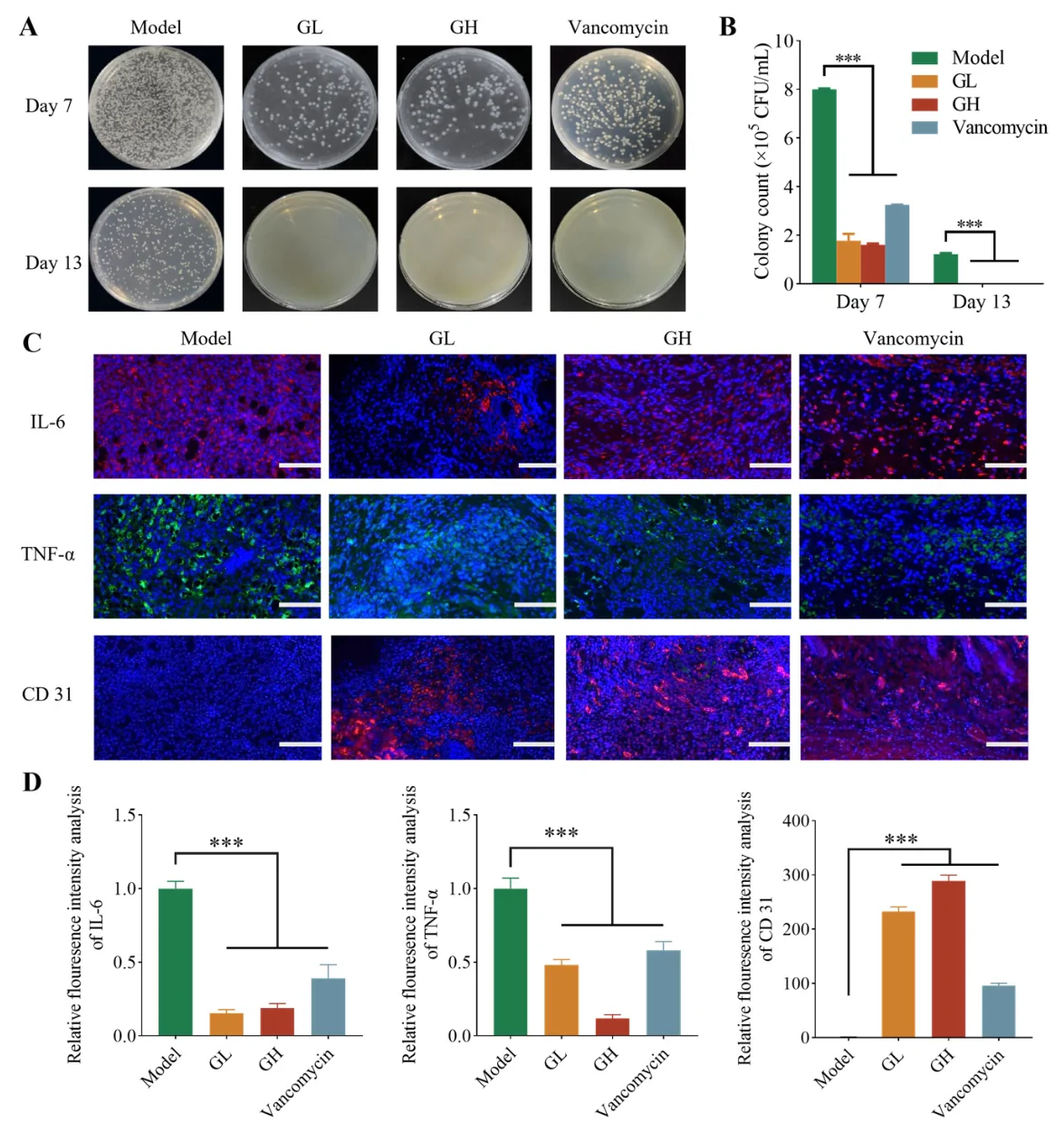
Promotion of diabetic wound healing by grifolin Via antibacterial, anti-inflammation and pro-angiogenesis effects. (**A**,**B**) Representative bacterial CFU images (**A**) recovered from MRSA-infected wound tissues on days 7 and 13, and corresponding statistical analysis (**B**) of CFU counts (*n* = 3). (**C**) Representative immunofluorescence images of wound tissue sections at day 7, showing the expression of IL-6 (red), TNF-α (green) and CD31 (red), with nuclei counterstained by DAPI (blue). Scale bar = 100 μm. (**D**) Relative fluorescence intensity analysis for IL-6, TNF-α and CD31 (*n* = 3). *** *p* < 0.001, compared with the model group.

Consistent with the observed histological improvements, grifolin promotes diabetic wound healing by eradicating bacterial infection and favorably modulating the wound microenvironment, thereby collaboratively addressing multiple barriers to tissue repair. First, grifolin can efficiently eliminate bacteria at the wound site. On days 7 and 13 of treatment, the bacterial load in wound tissues of grifolin-treated groups was significantly reduced compared with the model and vancomycin groups (Figure 6A,B), with the GH group showing the lowest CFU counts, controlling infection at its source and creating a favorable environment for healing.

Additionally, grifolin rectified the dysregulated wound immune microenvironment (Figure 6C,D). Immunofluorescence analysis revealed that grifolin treatment markedly decreased the expression of pro-inflammatory cytokines TNF-α and IL-6 at day 7 post-treatment, thus alleviating the excessive inflammatory state of diabetic wounds [47]. Simultaneously, grifolin strongly promoted angiogenesis, as evidenced by a significant increase in the fluorescence intensity of the vascular endothelial marker CD31. This enhancement of neovascularization improved local blood perfusion, facilitating the delivery of nutrients and oxygen to support tissue regeneration [48].

Furthermore, in vitro hemolysis assay exhibited that grifolin produced a low hemolysis rate at concentrations up to 100 μg/mL, suggesting acceptable blood compatibility (Supplementary Figure S5A). In the in vivo assessment, no overt systemic toxicity was observed at the therapeutic dose (Supplementary Figure S5B,C): body weights remained stable throughout treatment, and no drug-associated histopathological abnormalities were observed in the major organs. These findings indicate that grifolin was well tolerated at the therapeutic dose under the present experimental conditions, while a more comprehensive safety evaluation would be required before clinical translation.

## 4. Discussion

The rising prevalence of antimicrobial resistance has progressively eroded the effectiveness of conventional antibiotics, as resistant bacteria evolve diverse mechanisms that bypass, modify or destroy their drug targets. This problem is especially pressing for MRSA, a major cause of persistent and healthcare-associated infections, and it underscores an urgent need for alternative strategies and new antibacterial targets. In this setting, developing novel multitarget agents that are less susceptible to single-step resistance is among the most promising approaches. Grifolin, a natural phenolic compound we isolated from the edible mushroom *Albatrellus dispansus*, has attracted attention owing to its diverse bioactivities. The present study examines its bactericidal activity, anti-biofilm and antibiotic-sensitizing potential, underlying mechanism and in vivo therapeutic efficacy.

Grifolin acts as a rapid, concentration-dependent bactericidal agent with kinetics distinct from cell-wall synthesis inhibitors such as vancomycin. The leakage of cytoplasmic protein and uptake of propidium iodide point to loss of cytoplasmic membrane integrity, which is mechanistically credible because the membrane is indispensable for permeability, energy transduction and homeostasis [49,50]. Beyond MRSA ATCC 43300, grifolin was equally or more effective against methicillin-susceptible *S. aureus* and the hypervirulent USA300 strain. Its preferential activity against Gram-positive organisms is also consistent with this interpretation, as the outer membrane restricts access to the cytoplasmic membrane. Membrane-active molecules are attractive in an era of resistance because their target cannot readily be altered by a single mutation [51]. The present data, however, do not distinguish membrane disruption as the primary binding event from a downstream consequence, and this remains an open question.

The checkerboard findings show that grifolin can enhance conventional antibiotics, with synergistic interactions for some combinations and additive effects for others. A reasonable explanation is that increased membrane permeability may facilitate entry of co-administered drugs to their intracellular targets, lowering the effective concentration needed to suppress growth [52,53]. This is particularly relevant to β-lactam resistance in MRSA, where membrane-active adjuvants that re-sensitize resistant organisms and may permit dose reduction have emerged as a promising strategy [52,54].

The clinical relevance of any anti-MRSA agent depends on its activity against biofilms, whose EPS matrix and embedded slow-growing cells confer broad tolerance that conventional antibiotics penetrate poorly [55,56]. Grifolin reduced not only biofilm biomass but also the metabolic activity and recoverable viability of resident bacteria, and confocal imaging confirmed substantial structural disruption and loss of living cells. A membrane-directed mechanism offers a plausible explanation, because damaging the envelope of metabolically recalcitrant cells does not rely on active replication and can remain effective where cell-wall agents falter [49]. Whether grifolin additionally penetrates or directly dismantles the EPS, however, cannot be resolved from the current evidence.

The integrated transcriptomic and proteomic analyses provide a systems-level interpretation broadly coherent with the phenotypes. The dominant transcriptional signature—centered on ribosome and translation, together with changes in membrane, energy metabolism, transport, quorum sensing and virulence—was largely mirrored at the protein level, where membrane transport and protein-processing functions predominated and the two molecular layers changed in the same direction for most responsive genes. Network analysis further organized these changes into interconnected modules spanning amino-acid metabolism, iron acquisition and cell-surface anchoring in addition to translation. Such convergence supports a multi-target response rather than an effect on a single receptor, and attenuation of virulence and resistance-related programs is consistent with the membrane and susceptibility phenotypes [45]. These should be treated as coordinated downstream responses; however, omics data establish correlation but neither the primary target nor causal sequence. Discriminating the initiating event from secondary adaptation will require target-oriented biochemical and genetic experiments.

Moving to an infected diabetic wound model, grifolin translated its antibacterial activity into improved healing, illustrating how infection control and microenvironment remodeling act in concert. By reducing the bacterial burden, it removes a persistent inflammatory stimulus especially damaging in diabetic wounds, while attenuation of pro-inflammatory cytokines and enhancement of angiogenic markers indicate that it also helps resolve excessive inflammation and restore the vascular supply needed for repair. These interconnected effects are consistent with the view that treating infected chronic wounds requires addressing infection, inflammation and perfusion together [57,58,59]. Given that murine wounds differ from human diabetic wounds in anatomy, healing kinetics, and the resident microbiota [60], and that the local exposure, residence time, and tissue penetration of topically delivered grifolin have not been defined, the dose-exposure relationship underpinning efficacy and safety in patients remains to be established. Overall, grifolin emerges as a membrane-active, multitarget candidate with anti-biofilm and antibiotic-sensitizing properties, positioning it as a credible lead for further development against MRSA.

## 5. Conclusions

In summary, this study systematically evaluated the in vitro and in vivo anti-MRSA activity and mechanism of grifolin and its potential for diabetic wound infection. Grifolin rapidly killed MRSA by disrupting the cytoplasmic membrane, eradicated mature biofilms, and sensitized MRSA to conventional antibiotics. Integrated transcriptomic and proteomic analyses supported by qRT-PCR and PPI networks revealed a coherent multitarget response spanning translation, membrane and energy metabolism, quorum sensing and virulence. In a murine model of MRSA-infected diabetic wounds, grifolin significantly accelerated wound closure by reducing the bacterial burden, suppressing inflammation, promoting angiogenesis and enhancing collagen deposition. Collectively, grifolin is a membrane-damaging, multitarget lead that combines antibiofilm and antibiotic-sensitizing activities, offering a promising alternative to conventional antibiotics against MRSA and other resistant, biofilm-associated infections.

## Data Availability

The data that support the findings of this study are available from the corresponding author upon reasonable request.

## Supplementary Materials

The following supporting information can be downloaded at: https://www.mdpi.com/article/10.3390/biology15212019/s1, Supplementary Table S1: The MIC and MBC values for all tested strains. Supplementary Table S2: Combination of grifolin with different antibiotics. Supplementary Table S3: Primer sequences for qPCR. Supplementary Table S4. Expression stability (M value) of five candidate reference genes in MRSA evaluated by geNorm. Supplementary Figure S1. (A) Growth curves of MRSA following treatment with grifolin or vancomycin. (B) The viability of MRSA assessed by MTT assay. Data are expressed as mean ± SD (n = 3). Supplementary Figure S2. Antibacterial activity of grifolin against *S. aureus* and MRSA USA300. (A) OD_600_ growth curves of *S. aureus* and MRSA USA300 at the indicated grifolin concentrations. (B,C) Bacterial count (B) and representative colony images (C) of *S. aureus* and MRSA USA300 after treatment with grifolin (0, 1, 2, 4 µg/mL) or vancomycin (1 µg/mL). Data are expressed as mean ± SD (n = 3). Supplementary Figure S3. Expression stability of reference genes. (A) Ct value distribution of five reference genes in MRSA. (B) M values calculated by geNorm. Data are expressed as mean ± SD (n = 3). Supplementary Figure S4. (A) Correlation coefficient distribution plot. Each cell in the plot indicates the correlation coefficient calculated between the corresponding two samples based on protein quantification data, with color intensity reflecting the magnitude of the correlation coefficient. (B) Peptide count distribution plot. Supplementary Figure S5. Biosafety evaluation. (A) Hemolysis rates of different concentrations of grifolin on red blood cells. Insets: images of the corresponding red blood cells solutions. (B) Weight change of mice (n = 5). (C) H&E staining of heart, liver, spleen, lungs, and kidneys of mice. Scale bar = 100 μm.
