# Supplementary material for: Grifolin Exhibits Promising Antimicrobial Potential Against MRSA Infection
Source: Biology (Basel). 2026 Nov 6;15(21):2019. doi: 10.3390/biology15212019 (PMC13761891; doi:10.3390/biology15212019)
Supplement: Supplementary file 1 [file biology-15-02019-s001.zip › biology-4616930-supplementary.pdf]

Supplementary Material

Grifolin Exhibits Promising Antimicrobial Potential Against MRSA Infection

Guosheng Hu †, Yun Zhou †, Mengxi Wang, Rong Huang *, Jikai Liu*

The Modernization Engineering Technology Research Center of Ethnic Minority Medicine of Hubei Province, School of Pharmaceutical Sciences, South-Central Minzu University, Wuhan 430074, China

* **Correspondence:** ronghuang@mail.scuec.edu.cn (R.H.); liujikai@mail.scuec.edu.cn (J.L.)

† These authors have contributed equally to this work.

S1 Supplementary methods

S1.1 Transcriptomic analysis

RNA extraction and quality control: After 3 h of co-incubation at 37 ° C, bacterial cells were collected and total RNA was extracted. RNA integrity and genomic DNA contamination were assessed by agarose gel electrophoresis. RNA purity (OD260/280 and OD260/230 ratios) was evaluated using a Nanodrop One C (Thermo Fisher Scientific Inc., USA), and RNA concentration was quantified using a Qubit 2.0 Fluorometer (Invitrogen, Grand Island, NY, USA). RNA integrity was further verified using an Agilent 2100 Bioanalyzer (Agilent Technologies, Santa Clara, CA, USA).

Library preparation and sequencing: For each sample, 2 μg of total RNA was used for library construction with Illumina® Stranded mRNA Prep. mRNA with poly(A) tails was enriched using Oligo(dT) magnetic beads and fragmented using divalent cations in Fragmentation Buffer. The first-strand cDNA was synthesized using fragmented mRNA as a template and random oligonucleotides as primers in the M-MuLV reverse transcriptase system. The RNA strand was then degraded with RNase H, and second-strand cDNA was synthesized using DNA polymerase I and dNTPs. The double-stranded cDNA was purified, end-repaired, A-tailed, and ligated with sequencing adapters. cDNA fragments of approximately 200 bp were selected using AMPure XP beads, followed by PCR amplification and purification to obtain the final library. Qualified libraries were pooled and sequenced on an utilizing Illumina sequencers.

Data analysis: Raw reads were filtered using fastp to remove adapter-containing reads, reads with an N content exceeding 10%, and reads in which low-quality bases ($Q \leq 20$) accounted for more than 50% of the read length. The resulting clean reads were mapped to the reference genome using HISAT2. Gene expression levels were quantified as fragments per kilobase of transcript per million mapped reads (FPKM) using HTSeq. Differential expression analysis was performed using DESeq2, which applies size-factor normalization, with P-values adjusted by the Benjamini-Hochberg method. Genes with an adjusted $P\text{-value} < 0.05$ and an absolute fold change $|FC| > 1.5$ were defined as significantly differentially expressed genes (DEGs). Sample-level reproducibility was assessed by Pearson correlation analysis and principal component analysis (PCA), which confirmed high concordance among biological replicates. Gene Ontology (GO), Kyoto Encyclopedia of Genes and Genomes (KEGG) pathway enrichment, gene set enrichment analyses (GSEA) were performed using hypergeometric tests on the Majorbio Cloud Platform.

S1.2 Proteomics analysis

Protein sample preparation: The lysis buffer was added to the sample to a final concentration of 1% SDC/100 mM Tris-HCl (pH=8.5). The mixture was sonicated and centrifuged at 12000 g for 5 min. Protein

concentration of the supernatant was determined by BCA method. With the same amount of protein among different samples, the solution (1% SDC/100 mM Tris-HCl, pH 8.5) was used to fill all samples to the same volume. Protein reduction and alkylation were conducted with TCEP and CAA at 60° C for 30 min. SDC was diluted below 0.5% with the same volume of ddH_2O addition. Trypsin was added at a ratio of 1: 50 (enzyme: protein, w/w) for overnight digestion at 37° C. The next day, TFA was used to bring the pH down to 6.0 to end the digestion. After centrifugation (12000 g, 15 min), the supernatant was subjected to peptide purification using a self-made SDB-RPS desalting column. The peptide eluate was vacuum dried and stored at -20 ° C for later use.

LC-MS/MS analysis: All samples were analyzed on timsTOF Pro (Bruker Daltonics), a hybrid trapped ion mobility spectrometer (TIMS) quadrupole time-of-flight mass spectrometer. An UltiMate 3000 RSLCnano system (Thermo) was coupled to timsTOF Pro with a CaptiveSpray nano ion source (Bruker Daltonics). Peptide samples were injected into a C18 Trap column (75 μm*2 cm, 3 μm particle size, 100 Å pore size, Thermo), and separated in a reversed-phase C18 analytical column (75 μm*15 cm, 1.7 μm particle size, 100 Å pore size, IonOpticks). Mobile phase A (0.1% formic acid in water) and mobile phase B (0.1% formic acid in ACN) were used to establish the seperation gradient at a flow rate of 300 nL/min. The MS were operated in diaPASEF mode. The capillary voltage was set to 1500 V. The MS and MS/MS spectra were acquired from 100 to 1700 m/z. The ion mobility was scanned from 0.6 to 1.6 Vs/cm^2. The accumulation time and ramp time were set to 50 ms. The diaPASEF acquisition scheme was defined according to the m/z-ion mobility plane using the timsControl software (Bruker Daltonics). The collision energy was ramped linearly as a function of the mobility from 59 eV at 1/K0=1.6 Vs/cm^2 to 20 eV at 1/K0=0.6 Vs/cm^2.

Database search: DIA raw data were analyzed with DIA-NN (DIA-NN 2.2.0 Academia) in library-free mode. Spectra files were searched against the customized protein sequence database (2672 entries). Search parameters were mainly default and set as follows: Precursor ion generation options were enabled for in silico-predicted spectral library generation; Trypsin/P with maximum 2 missed cleavage; Carbamidomethyl on C as fixed modification; Oxidation on M and protein N-terminal acetylation as variable modifications; the mass and MS1 accuracy was set to 15 ppm; MBR was enabled; Heuristic protein inference was enabled; Precursor FDR was set to 1% for reliable identifications. Protein intensities were normalized with MaxLFQ algorithm.

Data analysis: The "pg_matrix.tsv" file from DIA-NN search results was used for quantitative analysis at the protein level. All subsequent bioinformatics analyses were conducted in the R statistical programming environment. Missing values were imputed by drawing from a random Gaussian-shaped distribution centered around the mass spectrometer's detection limit, applying a downshift of 1.8 standard deviations

and a width of 0.25 standard deviations. Proteins with valid quantitative values in at least 50% of the samples within any experimental group were retained for further analysis. Significantly differentially expressed proteins (DEPs) were determined based on the P-value from Student's t-test and fold change (|FC| > 1.5; P < 0.05). Functional annotations were performed using Gene Ontology (GO), the Kyoto Encyclopedia of Genes and Genomes (KEGG), EggNOG, Pfam, and UniProt subcellular localization databases. Fisher's exact test was employed to identify significantly enriched pathways among the regulated proteins. Protein-protein interaction networks were constructed and analyzed using the STRING database.

S2 Supplementary tables and figures

S2.1 Supplementary tables

Supplementary Table S1. The MIC and MBC values for all tested strains

Strain	MIC (μg/mL)	MBC (μg/mL)
MRSA ATCC 43300	3	4
MRSA USA300	2	2
Staphylococcus aureus ATCC 25923	2	2
Escherichia coli ATCC 25922	> 32	> 32

Supplementary Table S2. Combination of grifolin with different antibiotics

Antibiotic	MIC (μg/mL)	FIC_B	$FIC_{A\text{-}Grifolin}$	FICI	Effect
Oxacillin	16	1/4	1/256	0.254	synergistic
Ampicillin	128	1/2	1/256	0.504	additive
Cefotaxime	64	1/2	1/256	0.504	additive
Cefoxitin	8	1/2	1/4	0.750	additive
Vancomycin	1	1/8	1/4	0.375	synergistic

Supplementary Table S3. Primer sequences for qPCR

Gene symbol	Sequence (5′ to 3′)
clfB	F: GGAAGTGCTGATGGTGATT<br>R: TCTGAGTCTGAGTCGCTAT
moeA	F: AATCAGGTGCGGTTGTAG<br>R: CAGCGTCAGATTCAGTCA
agrA	F: TTGCCCTCGCAACTGATAAT<br>R:CAACTGGGTCATGCTTACGA
murQ	F: GTACTTCTGGTGCTAATGATAC<br>R: ACGCCATTGCTTCATCAT
vwb	F: GTGGTTTCTGGGGAGAAGAAT<br>R: TTATTTGCCATTATACTTTATT
mecA	F: TGGCAGACAAATTGGGTGGT<br>R: TGAAGCAACCATCGTTACGGA
icaD	F: ATGGTCAAGCCCAGACAGAG<br>R: AGTATTTTCAATGTTTAAAGCAA
rplN	F: CAGGTGTTCGTCGTAATGA<br>R: GTACTTCTGGTGCTAATGATAC
dltB	F: GGTTAGGTGGACATCAGATT<br>R: ACGATATTCATTGCCTGTTG
infC	F: CGGTCAAGATGGTGAACAA<br>R: ACTGGTGGTTTCGCATTC
16S rRNA	F: CGCAATGGGCGAAAGC<br>R: TACGATCCGAAGACCTTCATCA
gyrB	F: GGTGGCGACTTTGATCTAGC<br>R: TTATACAACGGTGGCTGTGC
rpoB	F:CAGCTGACGAAGAAGATAGCTATGT<br>R:ACTTCATCATCCATGAAACGACCAT
tuf	F:TCCTGGTTCAATTACACCACATACTG<br>R:GGAAATAGAATTGTGGACGATAGTTTGA
gapA	F:TGACACTATGCAAGGTCGTTTCAC<br>R:TCAGAACCGTCTAACTCTTGGTGG

Supplementary Table S4. Expression stability (M value) of five candidate reference genes in MRSA evaluated by geNorm.

Gene	Average Ct value of PBS (Mean ± SD)	Average Ct value of Grifolin (Mean ± SD)	Average Ct value of all samples (Mean ± SD)	M value
gyrB	20.0473 ± 0.0426	20.1032 ± 0.0082	20.0753± 0.0411	0.147
rpoB	20.0798 ± 0.0523	20.2507 ± 0.0775	20.1652± 0.1107	0.1666
16S rRNA	8.2426 ± 0.0490	8.1864 ± 0.1471	8.2145 ± 0.1028	0.1789
tuf	18.4937 ± 0.1148	18.6242 ± 0.1663	18.5590± 0.1464	0.1936
gapA	17.7451 ± 0.1042	17.4216 ± 0.0477	17.5833± 0.1914	0.2431

The relative quantity (Q_{ij}) and stability value (M_i) were calculated using the following formulas:

$$Q_{ij} = E^{(Ct_{\min,i} - Ct_{ij})}; \; M_i = Mean\left[SD\left(\left|\log_2 \frac{Q_{ik}}{Q_{jk}}\right|\right)\right]$$

Where E = amplification efficiency; Ct_{ij} = Ct value of gene i in sample j; $Ct_{\min,i}$= the minimum Ct value of gene i across all samples; Q_{ij} and Q_{ik} are the relative quantities of gene i and gene j in sample k, respectively.

S2.2 Supplementary figures

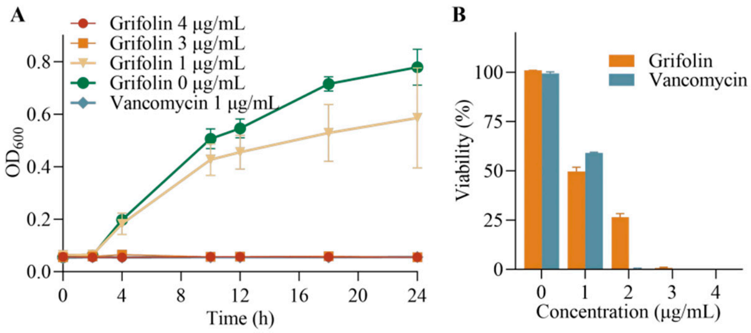

Supplementary Figure S1. (**A**) Growth curves of MRSA following treatment with grifolin or vancomycin. (**B**) The viability of MRSA assessed by MTT assay. Data are expressed as mean±SD (n = 3).

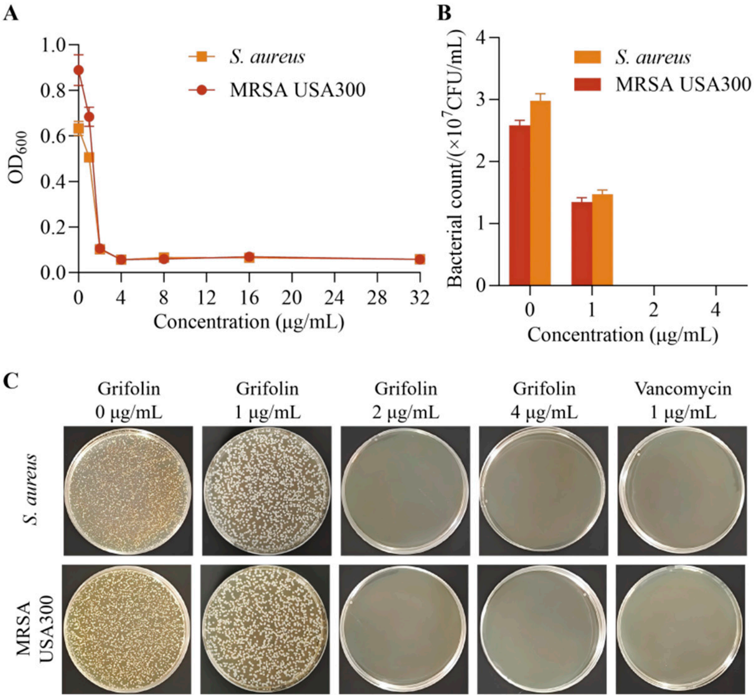

Supplementary Figure S2. Antibacterial activity of grifolin against *S. aureus* and MRSA USA300. (**A**) OD_{600} growth curves of *S. aureus* and MRSA USA300 at the indicated grifolin concentrations. (**B**,**C**) Bacterial count (B) and representative colony images (C) of *S. aureus* and MRSA USA300 after treatment with grifolin (0, 1, 2, 4 μg/mL) or vancomycin (1 μg/mL). Data are expressed as mean ± SD (n = 3).

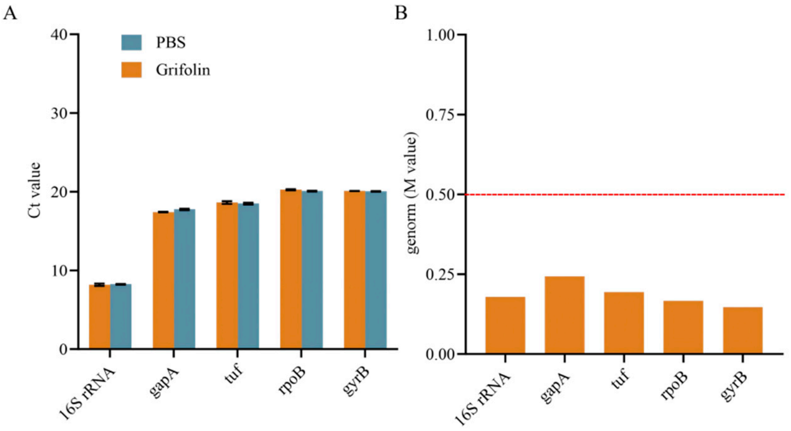

Supplementary Figure S3. Expression stability of reference genes. (**A**) Ct value distribution of five reference genes in MRSA. (**B**) M values calculated by geNorm. Data are expressed as mean±SD (n = 3).

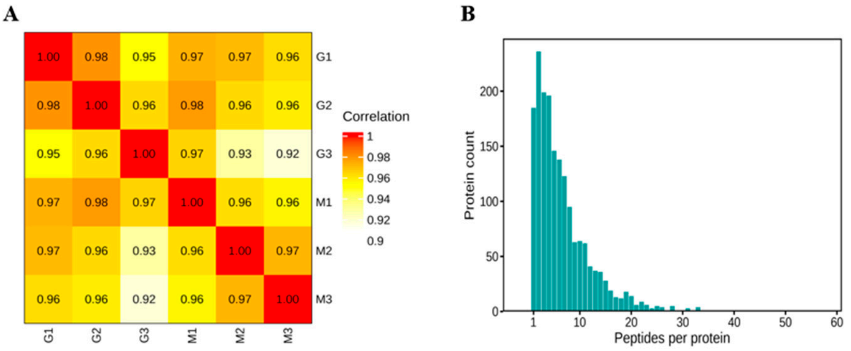

Supplementary Figure S4. (A) Correlation coefficient distribution plot. Each cell in the plot indicates the correlation coefficient calculated between the corresponding two samples based on protein quantification data, with color intensity reflecting the magnitude of the correlation coefficient. (B) Peptide count distribution plot.

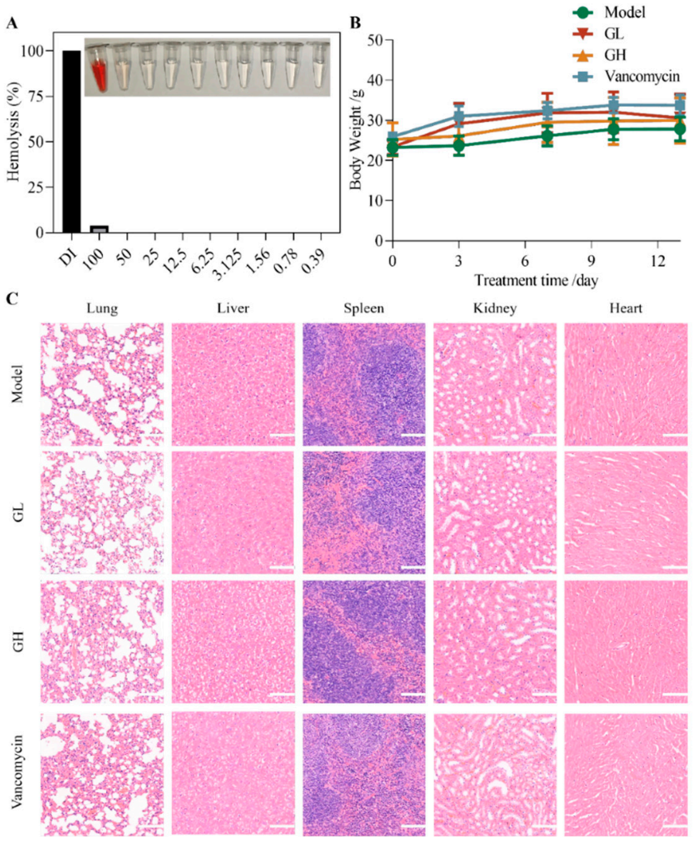

Supplementary Figure S5. Biosafety evaluation. (**A**) Hemolysis rates of different concentrations of grifolin on red blood cells. Insets: images of the corresponding red blood cells solutions. (**B**) Weight change of mice (n = 5). (**C**) H&E staining of heart, liver, spleen, lungs, and kidneys of mice. Scale bar=100 μm.
